# A Methodology for Sustainable Management of Food Waste

**DOI:** 10.1007/s12649-016-9720-0

**Published:** 2016-10-25

**Authors:** Guillermo Garcia-Garcia, Elliot Woolley, Shahin Rahimifard, James Colwill, Rod White, Louise Needham

**Affiliations:** 1 Centre for Sustainable Manufacturing and Recycling Technologies (SMART), Loughborough University, Loughborough, Leicestershire UK; 2 Molson Coors, Burton-on-Trent, Staffordshire UK; 3 Quorn Foods, Stokesley, North Yorkshire UK

**Keywords:** Food waste, Waste categorization, Waste management, Food sustainability, Brewery waste, Mycoprotein waste

## Abstract

As much as one-third of the food intentionally grown for human consumption is never consumed and is therefore wasted, with significant environmental, social and economic ramifications. An increasing number of publications in this area currently consider different aspects of this critical issue, and generally focus on proactive approaches to reduce food waste, or reactive solutions for more efficient waste management. In this context, this paper takes a holistic approach with the aim of achieving a better understanding of the different types of food waste, and using this knowledge to support informed decisions for more sustainable management of food waste. With this aim, existing food waste categorizations are reviewed and their usefulness are analysed. A systematic methodology to identify types of food waste through a nine-stage categorization is used in conjunction with a version of the waste hierarchy applied to food products. For each type of food waste characterized, a set of waste management alternatives are suggested in order to minimize environmental impacts and maximize social and economic benefits. This decision-support process is demonstrated for two case studies from the UK food manufacturing sector. As a result, types of food waste which could be managed in a more sustainable manner are identified and recommendations are given. The applicability of the categorisation process for industrial food waste management is discussed.

## Introduction

Food waste is one of the most challenging issues humankind is currently facing worldwide. Currently, food systems are extremely inefficient: it is estimated that between one-third and one half of the food produced is lost before reaching a human mouth [1, 2]. The Sustainable Development Goal 12 ‘Ensure sustainable consumption and production patterns’ established by the United Nations in 2015 includes a specific target for food waste reduction: halve per capita global food waste at retail and consumer levels by 2030. Additionally, it also includes a more general goal to reduce food losses along food supply chains [3]. Therefore, it is expected that there will be an increasing number of initiatives, campaigns and legislative developments in order to reach the aforementioned objectives.

Nevertheless, reduction of the current levels of food waste must be accompanied by better management of the waste: inevitably there will always be some food waste. Furthermore, some parts of the food products are inedible and will unavoidably become a waste stream. There are countless alternatives to manage food waste, however the most common solution worldwide is still landfilling [4], which is highly damaging to the environment and poses a risk to human health, whereas it does not provide any benefit. In spite of the progress achieved in recent years to find alternative solutions, particularly in developed nations, better management of food waste in supply chains is still required.

Sustainable management of food waste is a momentous research area that has rapidly grown over recent years. Meritorious examples of research aiming to find sustainable solutions for food waste management are numerous, but they have been generally inclined to look into only one area of sustainability: environmental, economic or social ramifications [5, 6]. Recent research aims to expand the scope and consider two or even all three pillars of sustainability implications mentioned above. Remarkable examples are work by Münster et al. [7], Ahamed et al. [8] and Martinez-Sanchez et al. [9], who consider economic and environmental ramifications of food waste management.

Nevertheless, as the scope of this research area expands, systematic analyses are needed to obtain comparable results. Examples of frameworks with this aim have been developed for solid waste management (e.g. [10, 11]), but are less common for food waste management. A recent example of this is the framework recently developed by Manfredi et al. [12], which provides a useful six-step methodology to evaluate environmental and economic sustainability of different alternatives to manage food waste, with the aim of also incorporating social considerations.

The waste hierarchy applied to food products is a useful tool to rank waste management alternatives by sustainability performance. The waste hierarchy concept was introduced for the first time into European waste policy in 1975 [13], and has been continuously used until today in European Directives which have been implemented since then. It is also used in the UK by the Government and institutions such as Defra [14] and WRAP [15], and has been implemented in UK law [16]. There is a considerable number of research papers published in prestigious scientific journals discussing the waste hierarchy, plenty of them focussed on food waste, e.g. [17, 18]. More detailed information on the technologies described in the food waste hierarchy and their associated emissions can be found in the Best Available Techniques for the Waste Treatments Industries [19].

This paper describes a novel, systematic methodology to support sustainable decisions regarding management of food waste. With this objective, a nine-stage categorization and a version of the food waste hierarchy are used as a basis of a methodical procedure to identify types of food waste and alternative activities to manage them. As a result, a novel Food Waste Management Decision Tree is developed and discussed, and its applicability is tested using two case studies from the UK food manufacturing sector.

## Methodology

### Research Aim and Structure

**Fig. 1:**
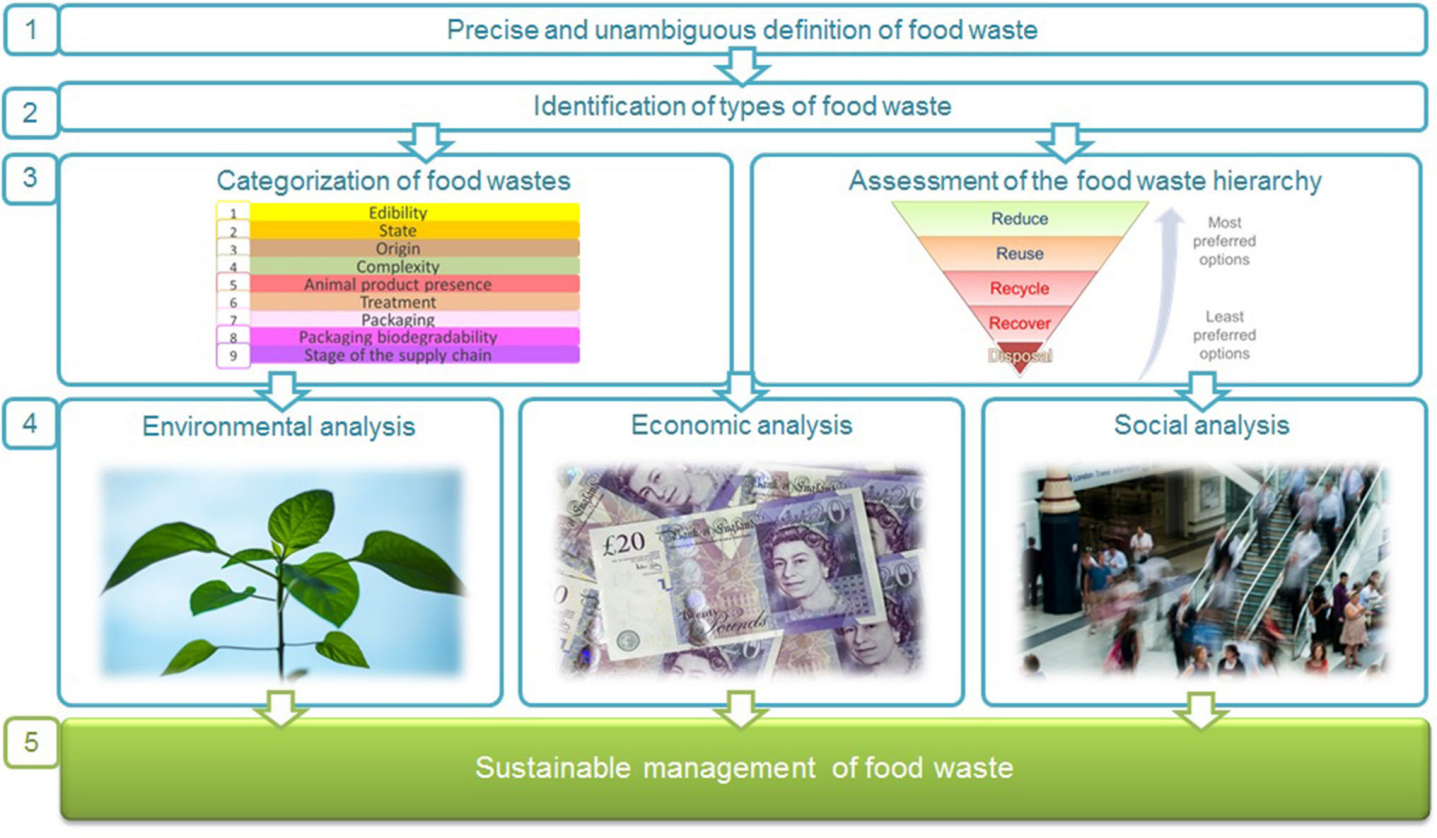
Structure of the research presented in this paper

The decision as to which is the most beneficial waste management alternative to utilise to manage food waste is usually made considering fundamentally only economic reasons and availability of waste management facilities. Furthermore, legislation delimits the range of solutions applicable to manage different types of food waste and therefore the decision is often made considering only a few alternatives. This paper seeks to add environmental and social considerations to the decision-making process so that more sustainable solutions can be achieved from the range of feasible waste management options. With this aim, the structure of the research presented in this paper is as follows: firstly, the definition of food waste used throughout this paper is provided; secondly, previous categorizations of food waste are discussed; thirdly, a categorization process is described based on the most pertinent indicators to classify food wastes; fourthly, the different types of food waste identified are linked to their most appropriate waste management alternatives, building a Food Waste Management Decision Tree; and finally, the categorization process is illustrated with two case studies from the UK food industry. A visual model of the research approach used can be seen in Fig. 1.

### Definition of Food Waste

The first aspect to look upon in order to improve food waste management is to define unambiguously the exact meaning of ‘food waste’. Unfortunately an agreement has not been reached yet and rather there are a range of definitions used. For consistency in this paper, food waste will be defined as food materials (including drinks) originally intended to be used to feed humans and not ultimately sold for human consumption by the food business under study, and inedible parts of food. Consequently, food sent to charities by companies is considered food waste in this paper, as it implies an economic loss to the food business, although from a biological and legal aspect this product remains being food and could be classified as surplus food. Inedible parts of food are also included in the definition because waste is often composed of both edible and inedible parts difficult to separate, and food businesses must manage this waste. Inedible food waste is thus considered unavoidable waste. Any food used in other way than for human consumption is also considered food waste (e.g. animal feeding, industrial uses). On the other hand, food wasted by consumers and managed at home (e.g. home composting) falls out of the scope of this paper. Clearly, the inclusion of these factors in the definition is debatable; this paper studies the management of these materials and therefore they have been included in the term ‘food waste’.

### Review on Methods to Classify Food Waste

Categorization is a key step in order to identify the most appropriate waste management alternative for different types of food waste. Such categorization should consider all the divisions necessary to link different types of food waste with treatment methodologies in a way that their economic and social benefit are maximised and their environmental impact is minimized. Usually different studies use their own categorizations [20]. This section describes different attempts to classify food waste. These classifications are assessed and their usefulness to select optimal food waste management alternatives is discussed.

The most obvious categorization divides different types of food waste according to the type of food: cereals, fruits, meat, fish, drinks, etc. This categorization is useful to quantify the amount of food wasted based on mass (more commonly), energy content, economic cost, etc. There exist plenty of examples to classify food waste according to its food sector, e.g. [21, 22]. This type of classification is typically based on codes, e.g. the recently published Food Loss and Waste Accounting and Reporting Standard recommends the use of the Codex Alimentarius General Standard for Food Additives (GSFA) system or the United Nations’ Central Product Classification (CPC) system as main codes, and when more precise classifications are needed, the Global Product Category (GPC) code or the United Nations Standard Products and Services Code (UNSPSC) as additional codes [23]. Additionally, food waste can be categorized with regard to its nutrient composition (e.g. carbohydrate and fat content [24]), chemical composition (e.g. C, H, N, O, S and Cl content [25]) or storage temperature (e.g. ambient, chilled or frozen [26]). Nonetheless, the information provided with these examples is not enough to prioritise some waste management alternatives against others.

In the UK, WRAP also identified the stages of the supply chain where food waste was generated (e.g. manufacturer, retailer) and assess the edibility of the waste. In this way, food waste can be avoidable (parts of the food that were actually edible), unavoidable (inedible parts of the food, such as bones, fruit skin, etc.) and possibly avoidable (food that some people would have eaten and others do not, such as bread crusts and potato skins) [27]. Different authors have further classified food waste at the household level as cooked/uncooked, as unpackaged/packaged food waste (when waste is packaged, it is additionally sorted as opened/unopened packaging) and according to their reason to disposal [28–30]. Other researchers also identified the leftovers and untouched food which goes to waste (e.g. [31]). Considering these options will be useful for a more comprehensive categorization, but there is still a lack of sections that further classify the waste in a way that a selection of the most appropriate waste management practice is facilitated. Furthermore, some of these classifications have been applied only to household food waste: a comprehensive categorization must include all stages of the food supply chain.

A more detailed attempt to classify food waste was carried out by Lin et al. [32], where food waste falls into the following categories: organic crop residue (including fruits and vegetables), catering waste, animal by-products, packaging, mixed food waste and domestic waste. In this study the potential for valorisation and some of the most appropriate options to manage the waste were assessed for each type of waste. However, the edibility of the waste and whether the food was fully processed during manufacturing were not considered.

Edjabou et al. [33] included two new factors: vegetable/animal-derived food waste and avoidable-processed/avoidable-unprocessed food waste. A more explicit classification with sub-categories was also suggested by Lebersorger and Schneider [20]. However the new sub-categories introduced, namely life cycle stage and packaging, are applicable only at the retail and household levels. They are irrelevant to improve the management of waste at other stages of the supply chain. On the other hand, Chabada et al. [34] used the ‘seven wastes’ approach from lean theory (namely transport, inventory, motion, waiting, overproduction, over-processing and defects) to classify categories of waste in fresh foods and identify the causes of waste generation, but not solutions for waste management. Garcia-Garcia et al. [35] suggested a number of indicators to classify food waste that provides useful information to delimit the range of waste management solutions applicable, nevertheless these indicators have not been used yet to identify the different types of food waste and propose the most appropriate waste management alternatives to manage them.

Therefore, a comprehensive and exhaustive analysis of all types of food waste has yet to be published. A holistic approach, where all relevant sub-categories of food wastes are identified and assessed, is necessary to support effective waste management. A solution to fill this knowledge gap is described in the following sections of this paper.

### Indicators to Classify Food Waste

The previous section of the paper highlights the lack of a standardised and holistic approach to food waste management and the need for a classification process applicable to all types of food wastes as defined previously. The final aim of such a classification is to provide support for a better selection of alternatives to manage food waste. Any scheme should allow prioritisation of sustainability decisions in terms of the three pillars of sustainability:

- Economic ramifications, which can be either positive (economic benefit obtained from management of the waste) or negative (economic cost to dispose of the waste).
- Environmental impacts, which are usually negative (e.g. greenhouse gas emissions), but can also be positive (e.g. use of waste for the removal of pollutants in wastewater).
- Social considerations, which can be either positive (e.g. food redistributed to people in need) or negative (e.g. increased taxes).

**Fig. 2:**
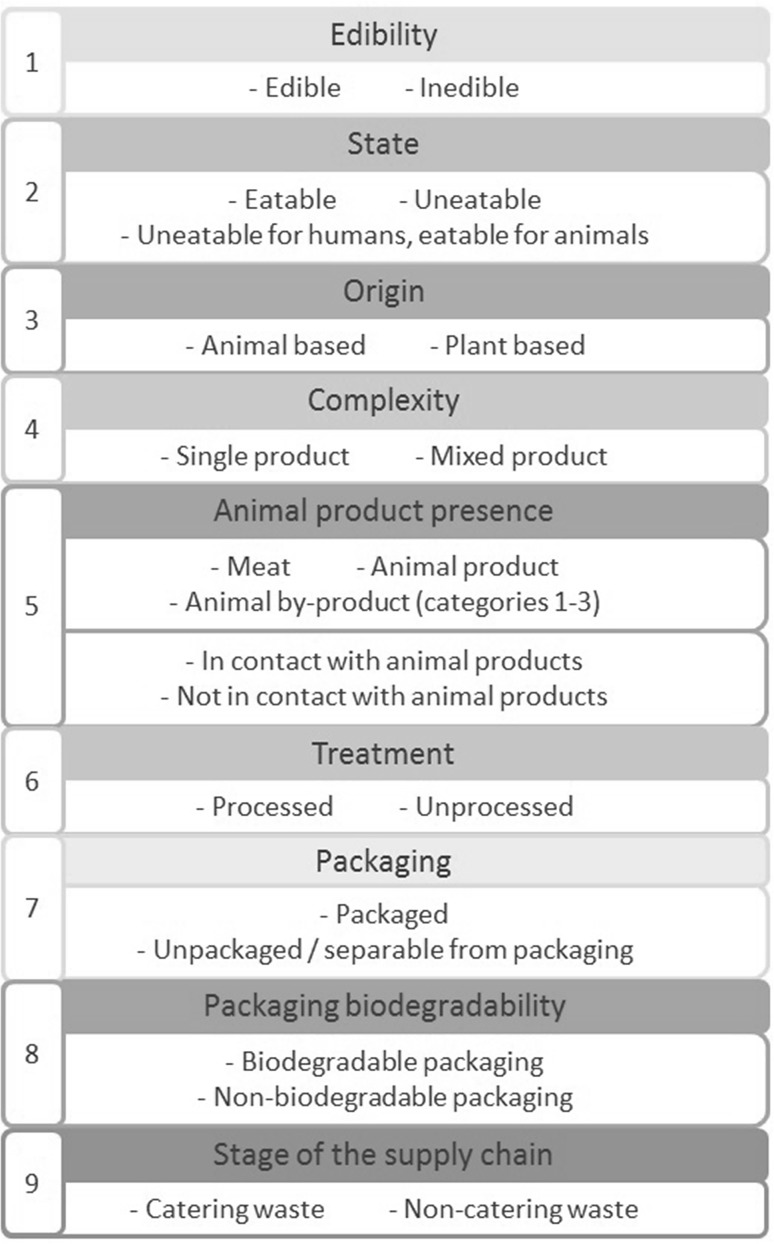
Indicators to categorize food waste. Adapted from Garcia-Garcia et al. [35]

The categorization proposed in this paper is based on nine indicators as explained by Garcia-Garcia et al. [35] and shown in Fig. 2. The assessment of these characteristics provides a systematic classification of the different types of food waste that enables a more appropriate selection amongst the available waste management alternatives. In each stage of the categorization process, one characteristic out of two or three options must be selected. Clarification of the different indicators can be found below:

1. *Edibility*: the product is edible if it is or has been expected to be consumed by humans at any point during its life cycle, otherwise the product is inedible. Inedible products include fruit skins, meat bones, some vegetable stalks, etc. When the product is edible from a biological point of view, but there is no demand for it (e.g. some types of offal, spent grain from breweries) it is considered inedible in this scheme, as it is not possible to reallocate it for human consumption. Therefore, the edibility of some food wastes can vary over time and geographical area considered. Various foods contain inedible parts when they are sold (e.g. banana and its skin); these food products are considered edible.
2. *State*: this characteristic must be assessed only for edible products. The product is eatable if it has not lost the required properties to be sold and fit for human consumption at the moment of its management as waste, otherwise the product is uneatable. If the food had not lost those properties, but requires further processing in the factory before being sold or consumed, it is classified as eatable and unprocessed (see indicator 6). A food product can become uneatable by being damaged at different points of the supply chain (e.g. overcooked during its manufacture, spilled during its distribution), being spoiled (e.g. leaving the cold chain), passing its use-by date, etc. If a product contains both uneatable and eatable parts and it is going to be managed as a whole, it must be considered uneatable. When the product is eatable from a biological point of view, there may still be ethical issues that can lead to classify it as uneatable to restrict its usage for human consumption, for instance to prevent using surplus alcoholic drinks for redistribution to charities, or products of lower quality to an acceptable established level. A third category includes products uneatable for humans because of safety concerns, but still fit for animal feeding (e.g. fallen from conveyor belts during manufacturing).
3. *Origin*: the product is animal based if it was produced by an animal (e.g. dairy products, eggs, honey) or using parts of animals (meat, including fish), otherwise the product is plant based. When the product contains both plant and animal-based materials (e.g. ready meals), it must be classified according to its predominant ingredient. If this is a plant ingredient the product will be also classified as a mixed product (see next categorization stage).
4. *Complexity*: this characteristic is only required for plant-based products. The product is single if it is formed of only one type of ingredient and it has not been in contact with other food material, otherwise the product is mixed.
5. *Animal product presence*: when the product is animal based, it must be categorized as meat (including fish), animal product (a product produced by animals) or by-product from animal bodies not intended for human consumption (e.g. by-products from slaughterhouses). In the last case, the waste should be further classified according to European regulations into Category 1, 2 or 3 [36]. When the product is plant based and mixed, it must be assessed as to whether the product contains any animal-based material or has been in contact with animal-based material.
6. *Treatment*: a food is considered processed when it has the same properties as the final product to be sold to the consumer (i.e. it has completed the manufacturing process, e.g. a ready meal; or the food does not need any processing before being distributed, e.g. fresh fruits and vegetables). If the food still needed any treatment at the moment of its management as waste it is unprocessed. Consequently, only edible and eatable waste should be assessed in this stage.
7. *Packaging*: a product is unpackaged if it is not contained in any packaging material. If the product is packaged but there is an available technology for unpacking and separating the food waste from its packaging, the product can be considered unpackaged; otherwise the product is packaged.
8. *Packaging biodegradability*: this characteristic must be assessed for packaged foods. Commonly, biodegradability of a material means that it can be digested by microorganisms, although the process may last for several months or years. Therefore, in this paper biodegradable packaging refers to that made of materials which have been tested and received a certificate of being “suitable for anaerobic digestion” or “compostable” in a technical composting plant (e.g. ‘DIN CERTCO’ logo and the ‘OK compost’ logo). Biodegradable packaging is generally composed of paper, bioplastics, wood or any plant-based product. Typically non-biodegradable packaging is made of plastic, glass or metal.
9. *Stage of the supply chain*: catering waste includes domestic waste and waste from food services (e.g. restaurants, schools, hospitals, etc.); non-catering waste is generated in earlier stages of the supply chain (i.e. during farming, manufacturing, distribution or retailing).

The assessment of these nine stages, and the consequent determination of nine characteristics, is the starting point to select the most convenient waste management alternative. The hypothesis of this work is that each combination of nine indicators has associated with it one most favourable solution. The nine-stage categorization scheme is intended to be easy to apply and determinative for selection of the optimal waste management alternatives, taking into account regulations and economic, environmental and social ramifications. The next chapter proposes a set of waste management alternatives for the different food waste types identified following the categorization based on the nine indicators explained in this section.

## Development and Partial Results

**Fig. 3:**
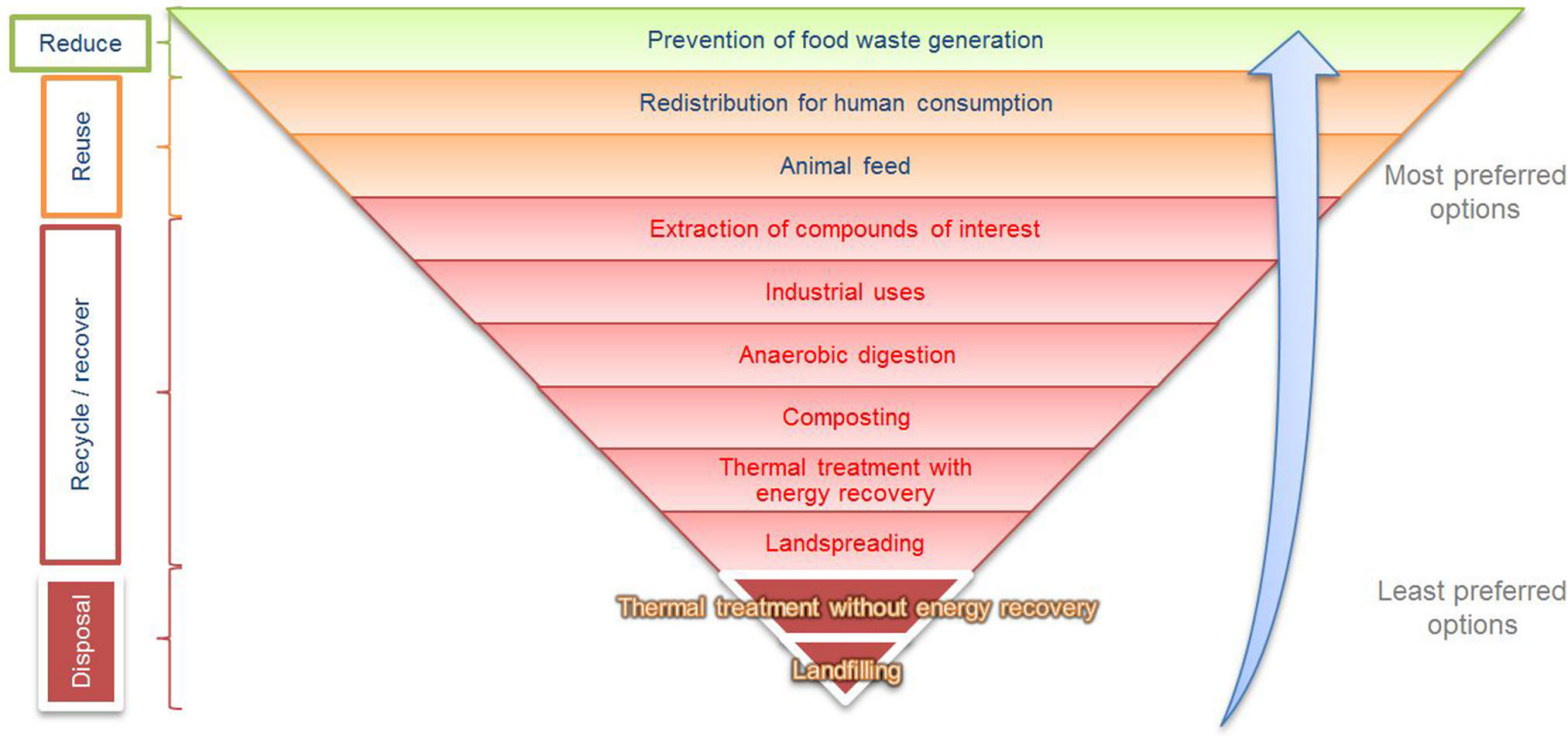
Waste hierarchy for surplus food and food waste. Adapted from Garcia-Garcia et al. [35] and based on Defra et al. [14], Adenso-Diaz and Mena [38], Papargyropoulou et al. [17] and Eriksson et al. [18]

Having identified and classified the different food wastes following the guidelines presented in the previous section, the next step is to identify and analyse the food waste management alternatives. In order to do so, the waste hierarchy applied to food products is an appropriate tool to classify the different options to manage food waste, based on the sustainability of its results. The particular order of the different options in the hierarchy (i.e. the preference of some alternatives against others) is debatable (e.g. anaerobic digestion is considered better than composting), but the final aim is to prioritize options with better environmental, economic and social outcomes. Hence, there are several slightly different adaptations of the food waste hierarchy, however the most recent versions are usually based on the Waste Framework Directive 2008/98/EC [37]. An example of a food waste hierarchy which aims to prioritise sustainable management alternatives can be seen in Fig. 3; it is based on previous versions, including those of Defra et al. [14], Adenso-Diaz and Mena [38], Papargyropoulou et al. [17] and Eriksson et al. [18].

It is difficult to apply a waste hierarchy to food products due to the heterogeneity of these materials and the numbers of actors at different stages of the food supply chain that waste food. Therefore, the waste hierarchy must be assessed for each type of food waste, rather than for ‘food waste’ as a whole. This case-specific application of the waste hierarchy has been also recommended by Rossi et al. in their analysis of the applicability of the waste hierarchy for dry biodegradable packaging [39].

In this paper, environmental, economic and social ramifications associated with food waste management are considered, but impacts of the food during its life cycle are not included as they do not affect food waste management decisions (i.e. the impacts have already occurred before the food was wasted). Consequently, a life-cycle approach was not necessary to assess different alternatives and only end-of-life impacts were studied.

**Fig. 4:**
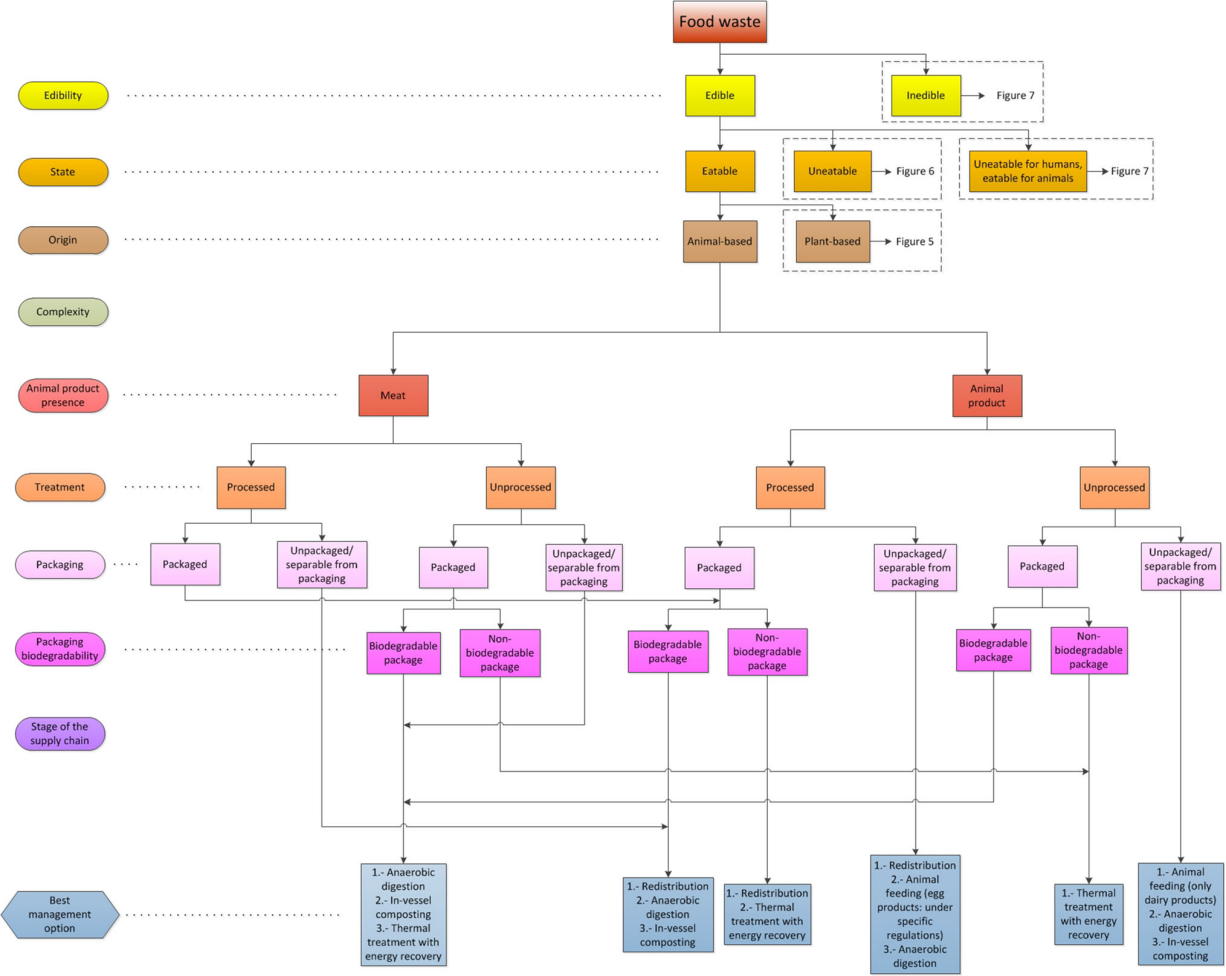
Food Waste Management Decision Tree (FWMDT). Edible, eatable, animal-based food wastes and their most convenient waste management alternatives

**Fig. 5:**
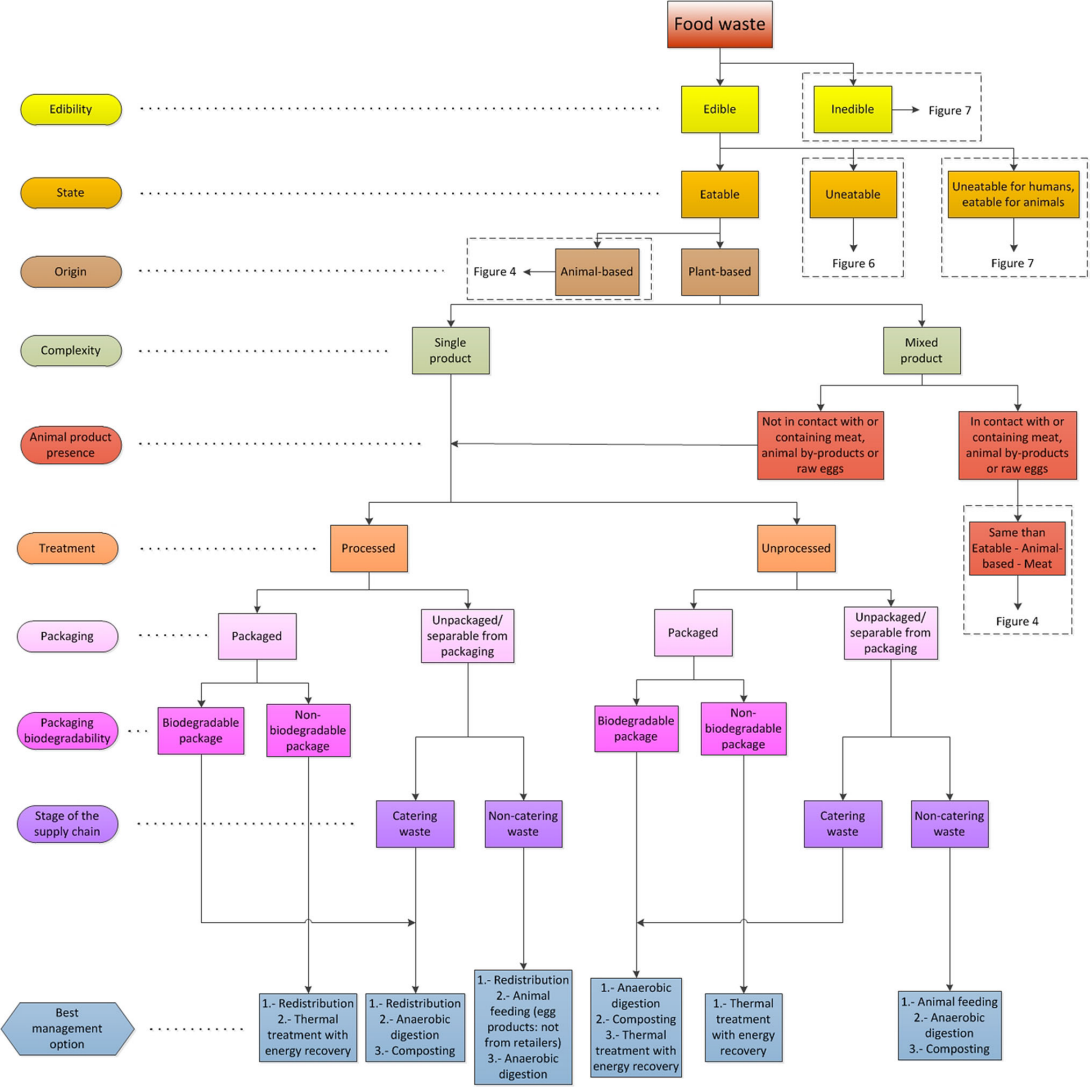
Food Waste Management Decision Tree (FWMDT). Edible, eatable, plant-based food wastes and their most convenient waste management alternatives

**Fig. 6:**
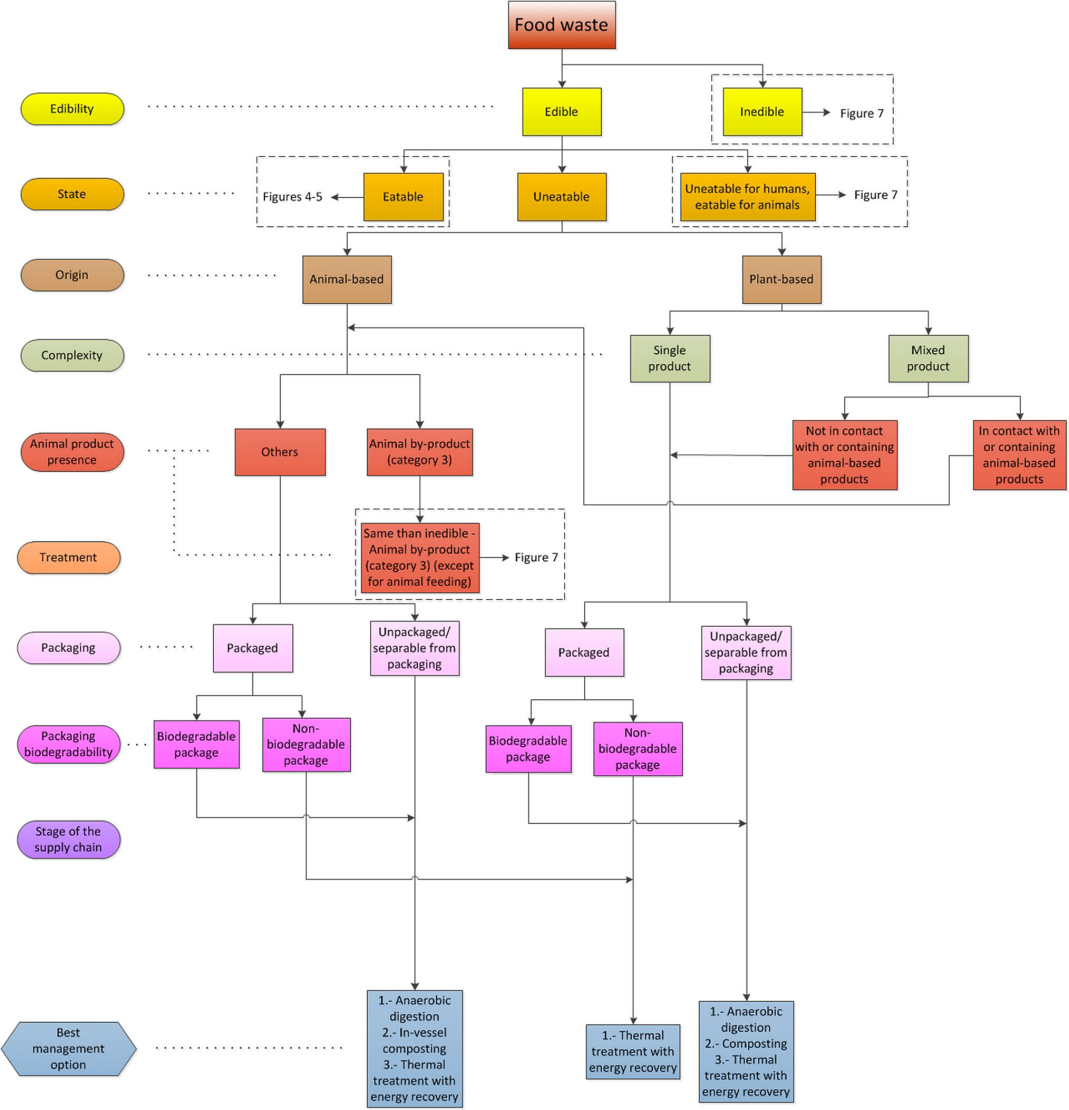
Food Waste Management Decision Tree (FWMDT). Edible, uneatable food wastes and their most convenient waste management alternatives

**Fig. 7:**
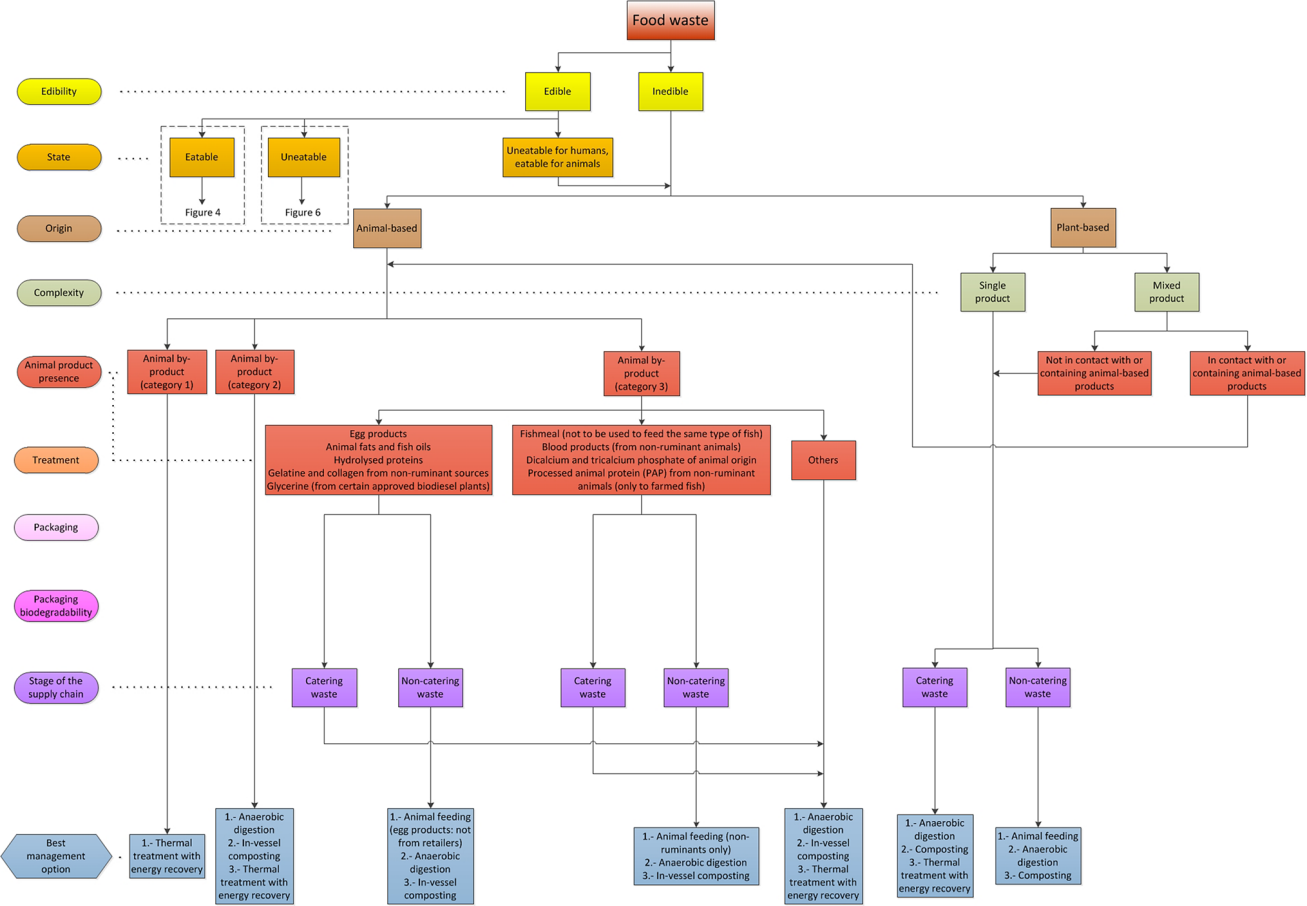
Food Waste Management Decision Tree (FWMDT). Inedible and uneatable for humans, eatable for animals food wastes and their most convenient waste management alternatives. The list of materials classified as animal by-products categories 1–3 can be found in [36]

In order to link the categorization process and the waste management alternatives from the food waste hierarchy, the indicators described previously have been firstly used to identify the different types of food waste. Each indicator has been assessed and the superfluous categories for each indicator have been eliminated to simplify the analysis (e.g. state for inedible waste). The optimal waste management alternatives have been identified for each type of food waste in compliance with UK and European regulations and based on the food waste hierarchy, therefore prioritising the most sustainable solutions (Fig. 3). The result of this analysis has been represented in a diagram (namely Food Waste Management Decision Tree, FWMDT) that helps with analysing food waste using the indicators described. This FWMDT has been divided into four parts for display purposes and can be seen in Fig. 4 (edible, eatable animal-based food waste), Fig. 5 (edible, eatable, plant-based food waste), Fig. 6 (edible, uneatable food waste) and Fig. 7 (inedible and uneatable for humans, eatable for animals food waste).

The FWMDT functions as a flowchart. The user begins at the highest level, and selects the indicator that best describes the food waste (e.g. edible or inedible). The user then moves through subsequent levels of the diagram, following the arrows and making further indicator selections. At the bottom the user is presented with a set of waste management alternatives that differ according to the set of indicators for that food type.

The food waste must be broken down for analysis into the same subgroups as for the treatments to be applied, e.g. if a food business generates both plant-based waste and animal-based waste which are collected and treated separately, they must be also assessed independently. However, if a producer of convenience foods produces undifferentiated waste composed of both plant and animal products, this must be studied as a whole. In the latter example, the waste is classified as a mixed product. It is readily seen that separate collection provides the benefit that more targeted management practices can be carried out on the different food waste streams. When separate collection is not possible, a thorough waste sorting is still recommended, although some of the alternatives will not be available then (e.g. plant-based food waste that has been in contact with meat cannot be used for animal feeding).

The development of a categorization that covers all types of food waste is arduous due to the number of waste types and their dissimilarity. Similarly, there are numerous alternatives for food waste management. In Fig. 3 some of these numerous alternatives have been grouped—for instance, all processes for extracting substances from all types of food waste are included in extraction of compounds of interest. This is because there are dozens of chemical and physical routes to obtain bio-compounds from food products, and also numerous possibilities to use different types of food waste for industrial applications such as removal of pollutants from wastewater. It is therefore unfeasible to consider all these options explicitly for all the food waste categories. Consequently, in all cases when there are management alternatives other than redistribution and animal feeding suggested in the FWMDT, a targeted study for each type of waste must be carried out in order to find what opportunities there are to extract compounds of interest or for industrial use, before considering options lower down in the food waste hierarchy.

Additionally, prevention of food waste generation is not included in the FWMDT because is out of the scope of this research, and also this option would be always prioritised, as it is at the top of the food waste hierarchy and can potentially be applied to all types of edible food wastes. The option of prevention also includes alternative uses of products for human consumption (e.g. a misshapen vegetable that can be used in convenience foods). In these cases the products must be reprocessed and they would not be considered food waste according to the definition provided in the previous section, and therefore they are out of the scope of this work. If instead they are directly consumed without further processing the alternative to follow will be redistribution, although this will normally give a smaller economic benefit to the food company than selling them at their normal price. In this paper it is assumed that all prevention steps have been taken to minimize food waste generation, but nevertheless food waste is created and requires waste management optimisation.

Landspreading can be used with the majority of food waste types, but according to the food waste hierarchy (Fig. 3) this alternative is less beneficial than composting. As both alternatives can be used to treat the same types of food wastes, landspreading has not been further considered in this work and only composting has been examined.

Additionally, the last two waste management practices, namely landfilling and thermal treatment without energy recovery, are not considered in the analysis. Landfilling has a high environmental impact, and its economic and social outcomes are also negative. Treatment without energy recovery damages the environment likewise, but its economic and social ramifications are generally less adverse. In both cases there are always more sustainable management practices that can be used to manage food waste, even if these two alternatives could be potentially used with all types of food waste, regardless of their nature.

The FWMDT was designed as far as possible to embody the categories and indicators described in the previous section, but this was not always achievable. For instance, the category animal-product presence includes additional indicators for inedible, animal-based products, as can be seen in Fig. 7, to comply with European regulations [36].

A description of each management alternative evaluated and the associated types of waste can be found below.

### Redistribution for Human Consumption

Redistribution for human consumption is the optimal alternative, as food is used to feed people. Agreements with charities and food banks help to distribute surplus food to those in need. Products must be edible, eatable and processed, as defined in the previous section. It must be noted that processed does not necessarily mean that the final product was fully processed as initially planned by the food business, e.g. surplus potatoes for the preparation of chips for ready meals can be redistributed if they are fit for human consumption and distribution (for example, they have not been peeled yet) and comply with regulations. In this case the potatoes are defined as processed because they are as sold to final consumers. The European legislation redistribution for human consumption must meet is the General Food Law [40], the Food Hygiene Package [41–44], the Regulation (EU) No 1169/2011 [45], and the Tax legislation [46], as explained by O’Connor et al. [47]. An extensive study of the situation of food banks and food donation in the UK was carried out by Downing et al. [48].

### Animal Feeding

This is the best alternative for foods which are not fit for human consumption but are suitable for animal feeding. In this category only farmed animals are considered (e.g. cattle, swine, sheep, poultry and fish). Pets, non-ruminant zoo animals, etc. are excluded, following guidelines explained in [49]. In order to be used for animal feeding, products must either be eatable or uneatable for humans but eatable for animals, unpackaged or separable from packaging, and non-catering waste. Inedible, plant based, single product, non-catering waste can be used for animal feeding depending on the type of waste. This particular case must be assessed for each type of waste independently. When the product is mixed, it must be either not in contact with or containing meat, by-products from animal bodies or raw eggs if it is eatable, or not in contact with or containing animal-based products if it is inedible or uneatable for humans but eatable for animals. Mixed waste containing animal products from manufacturers is suitable for animal feeding when the animal product is not the main ingredient. Meat (or plant-based products containing meat) cannot be sent for animal feeding. Eggs and egg products (or plant-based products containing them) must come from the agricultural or manufacturing stage when used for animal feeding and must follow specific treatments. Milk and dairy products can be used for animal feeding if they are processed (the processing needed is similar to that for human consumption), or unprocessed under UK rules if the farm is a registered milk processing establishment. Inedible, animal based, category 3 waste can also be used for animal feeding only under the conditions listed in the FWMDT (Fig. 7). According to European regulations, all types of category 3 animal by-products can be used in animal feed except hides, skins, hooves, feathers, wool, horns, hair, fur, adipose tissue and catering waste. Nevertheless the UK regulation is stricter than European regulations and this has been incorporated into the FWMDT. It must be noted that technically some category 3 animal by-products are edible, but they are not intended for human consumption. In any case, they must be not spoiled in order to be usable for animal feeding, and in most cases they must be processed following specific requirements before being used. If a waste contains different categories of animal by-products, it must be treated following the requirements of the material with the highest risk (category 1: highest risk, category 3: lowest risk). The following sources have been used to develop the FWMDT and must be consulted when using animal by-products in animal feeds: European regulations [36, 50, 51] and UK legislation [52]. Useful guidance information on this matter in the UK can be found at [49, 53]. Further information on additional legislation that applies to work with animal by-products can be found at [54] and [55] for milk products. Eggs must be treated in a processing facility under national rules [56]. The following additional legislation for animal feeding has also been consulted: European regulations [57–59] and regulations in England [60]. General guidance on animal feeding was collected by Food Standards Agency [61].

### Anaerobic Digestion

Anaerobic digestion can be used with all types of food waste except animal by-products category 1 and packaged waste (i.e. non-separable from packaging) in a non-biodegradable packaging. The animal by-products category 3 must be pasteurised; the particle size of animal by-products category 2 must be 50 mm or smaller, and its core must have reached a temperature of 133 °C for at least 20 min without interruption at an absolute pressure of at least 3 bar [36, 52, 62]. Anaerobic digestion plants in the UK must comply with regulations with regard to environmental protection, animal by-products, duty of care, health and safety and waste handling (more information about the different legal requirements can be found in [63]).

### Composting

The types of material suitable for composting are the same as for anaerobic digestion: all food waste except animal by-products category 1 and packaged waste (i.e. non-separable from packaging) in non-biodegradable packaging. Animal by-products category 2 can be composted if processed according to regulations [36, 52]. Composting must be carried out in closed vessels (in-vessel composting) if the waste contains or has been in contact with any animal-based material [15, 62], as it can attract vermin. Further guidance for the composting of waste can be found in [64].

### Thermal Treatment with Energy Recovery

This alternative can be applied to every type of food waste; nevertheless its use must be minimized as it provides small benefit compared to the impacts generated. Additionally, a great quantity of energy is needed to treat food waste due to its mainly high water content, and therefore this alternative may be useful and give an energy return on investment when treating dry food wastes (e.g. bread and pastries) or food waste mixed with other materials, such as in municipal solid waste. Thermal treatments with energy recovery, which includes incineration, pyrolysis and gasification, is the only alternative available to treat packaged food (non-separable from packaging) in non-biodegradable packaging, except the cases when the product is also edible, eatable and processed, and therefore can be redistributed for human consumption. As this type of waste is the final packaged product it will usually be generated in the last stages of the supply chain, particularly at retailing and consumer level (municipal solid waste). Thermal treatments with energy recovery are also the most appropriate alternative to treat animal by-products category 1, and in some cases, it is also necessary to process by pressure sterilisation [36, 52]. Useful information on incineration of municipal solid waste can be found in [65] and on technologies and emissions from waste incineration in the Best Available Techniques for Waste Incineration [66].

## Final Results and Discussion: Case Studies

### Introduction to Case Studies

The food waste categorization process presented in this paper has been applied to two case studies to demonstrate its applicability: a brewery (Molson Coors) and a manufacturer of meat-alternative products (Quorn Foods). These food companies were selected because previous contact between the researchers and the industries existed, and also due to their leading position in their product market, large size and therefore a predictable number of different types of food waste produced. A visit to their headquarters took place in June 2015, in which interviews were held with company employees. A questionnaire was used to systematically identify food waste streams and collect relevant data.

The categorization of these wastes according to the categorization scheme and the most favourable waste treatment alternatives identified using the FWMDT (Figs. 4–7) are explained in the following sections. The rest of the alternatives from the food waste hierarchy were also assessed for each type of food waste.

### Brewery: Molson Coors

This section categorizes the different types of food waste generated at one of Molson Coors’ manufacturing sites, a brewery situated in central England. The different types of food waste generated, in order of decreasing quantity, are: spent grain, waste beer, conditioning bottom, filter waste and trub. The quantity of waste generated during a year is only dependent on the level of production, since a relatively constant percentage of waste is generated per amount of final product manufactured. The different types of food waste identified are categorized in Table 1 and explained below.

**Table 1:** Types of food waste in Molson Coors and their management alternatives

	Spent grain	Waste beer	Conditioning bottom	Filter waste	Trub
Edibility	Inedible	Edible	Edible	Inedible	Inedible
State	N/A	Eatable	Eatable	N/A	N/A
Origin	Plant based	Plant based	Principally microorganisms*	Microorganisms*	Plant based
Complexity	Single product	Single product	Single product	Mixed product	Mixed product
Animal-product presence	N/A	N/A	N/A	Not in contact with animal-based products	Not in contact with animal-based products
Treatment	N/A	Processed	Unprocessed	N/A	N/A
Packaging	N/A	Separable from packaging	Unpackaged	N/A	N/A
Packaging biodegradability	N/A	N/A	N/A	N/A	N/A
Stage of the supply chain	Non-catering waste	Non-catering waste	Non-catering waste	Non-catering waste	Non-catering waste
Current treatment	Animal feeding	95 % animal feeding + 5 % sewage	Animal feeding	50 % compost + 50 % sewage	Animal feeding
Suggested alternative	Animal feeding	Redistribution for human consumption	Animal feeding	Anaerobic digestion	Animal feeding
Further possibilities	Production of foodstuff	N/A	Production of foodstuff	Industrial uses	Production of foodstuff
Quantity	≈70,000 t/year	14,000 t/year	7000 t/year	1200 t/year	≈700 t/year

The suggested alternative is based on the FWMDT presented in the Figs. 4–7. Possible alternative options from the food waste hierarchy are suggested as further possibilities when they are better than the suggested alternative. The particular type of diatomaceous earth in filter waste was not identified and thus it was considered to be not suitable for animal feeding. N/A means ‘not applicable’ or that the information is not necessary. * The ‘microorganisms’ indicator, from the origin stage, was considered as plant based

#### Spent Grain

Spent grain accounts for around 85 % of the total food waste in the manufacturing plant. It is an unavoidable by-product of the mashing process and is formed of barley and small amounts of wheat.

According to the FWMDT (Fig. 7), the best option is to send the waste for animal feeding. Currently spent grain is mixed with trub (in an approximate proportion of 99 % spent grain, 1 % trub) and used for animal feeding. However, the possibility of reprocessing the waste to adapt it for human consumption was also assessed, as suggested in the previous subsection. Spent grains contain high proportions of dietary fibres and proteins which may provide a number of health benefits [67]. Spent grain should not be mixed with trub if it is intended to use it to produce food products. Flour can be produced from spent grain following a process that includes drying and grinding [67]. This can be mixed afterwards with wheat flour and used in a wide range of food products such as bread, muffins, biscuits, etc., increasing their health benefits [68]. It must be noted that production of new food products was not selected by using the FWMDT because spent grain was considered inedible, as there is no current consumer demand for the products described above. If technology existed to produce new food products from spent grain, such as those described above, and these products could be sold because there was a consumer demand for it, spent grain would not be considered food waste providing it was used for this purpose.

Other uses for spent grain, apart from food uses and for animal fodder, include pet food, use in construction bricks, removal of pollutants in wastewater, production of paper, growing medium for mushrooms or microorganisms, extraction and synthesis of compounds (e.g. bioethanol, lactic acid, polymers and resins, hydroxycinnamic acids, arabinooligoxylosides, xylitol, pullulan), anaerobic digestion, composting, thermal treatment with energy recovery and landspreading [68–70].

#### Waste Beer

This waste corresponds to the final product which is not ultimately consumed. There are three reasons as to why this waste is generated:

- Beer left in casks brought back from the food service sector, which accounts for most of the waste in this category. It means an economic loss to the food service sector, not to the brewing company; therefore, it has not been given a high importance by the beer producer.
- Beer rejected because of mislabelling.
- Spilled beer in the filling process, which accounts for a negligible amount.

Currently, 95 % of the waste is sent to farms and mixed with other waste to feed animals (pigs). The remaining 5 % is sent to sewage.

Ideally, and according to the FWMDT (Fig. 5), beer left in casks could be reused for human consumption; however, as this comes from outside of the factory, it is difficult to prove that it has not been altered and is safe for consumption. If the option of redistribution for human consumption is discarded, the next recommended alternative is animal feeding, which is the current final use.

Beer rejected because of mislabelling is perfectly potable, so it is potentially reusable; however, there is difficulty of extracting the product from its packaging (i.e. emptying bottles and dispensing the product into new bottles). This would require significant employee time or new technologies for automation of the process, but would prevent beer from being wasted. Alternatively, in England the mislabelled beer can be sold at a lower price to a redistributor of surplus products such as Company Shop, where the label is corrected to meet Food Information Regulations 2014 [71], and providing the beer is compliant with food safety legislation it can be sold at a lower price to the final consumer. Similarly, European legislation that regulates the food information that must be provided to consumers in product labelling is the Regulation (EU) No 1169/2011 [45]. Food banks generally do not serve beer and therefore in these cases it cannot be redistributed to charities for people in need.

Alternatively, extraction of alcohol from waste beer by distillation could also give an economic benefit.

#### Conditioning Bottom

This waste is an unavoidable by-product which settles to the bottom of the conditioner tanks during the maturation process. It is composed principally of yeast, thus it is edible. However, it is not suitable for redistribution for human consumption, as the waste is not processed. Currently it is sent for animal feeding (pigs), which is the optimal alternative according to the FWMDT (Fig. 5).

Alternatively, some substances from the conditioning bottom can be used to produce new food products. Yeast can be separated and used to produce foodstuff. In order to recover yeast, the sediment should be filtered and squeezed, and this gives the opportunity to recover cloudy-type beer. As well as with spent grain, discussed previously, production of new food products was not selected by using the FWMDT because conditioning bottom is unprocessed, as there is either no current consumer demand for it or no technology available to undertake the processes required.

#### Filter Waste

Filter waste is formed of diatomaceous earth, yeasts and proteins. Yeast and proteins are edible; typically diatomaceous earth (i.e. fossilized remains of diatoms) is considered inedible; however there are two types: food grade diatomaceous earth and inedible diatomaceous earth. In order to choose the best waste management alternative the type of diatomaceous earth must first be identified. As the current use for beer production is as a filter medium, it will be assumed to be inedible diatomaceous earth.

Following the FWMDT (Fig. 7), the waste should be used in animal feeds. However, the type of diatomaceous earth used is not suitable for animal feeding and therefore the next alternative from the food waste hierarchy was suggested: anaerobic digestion to obtain energy. Currently, filter waste is sent to composting (when it is dry) and sewage (when it is wet). As composting is an alternative under anaerobic digestion in the waste hierarchy and sewage is at the bottom of the hierarchy, there is an important opportunity for improvement. Potential additional uses of diatomaceous earth include industrial (filter medium, stabiliser of nitroglycerin, abrasive in metal polishes and toothpaste, thermal insulator, reinforcing filler in plastics and rubber, anti-block in plastic films, support for catalysts, activation in blood coagulating studies, cat litter, etc.), additive in ceramic mass for the production of red bricks, insecticide and anticaking agent for grain storage (when it is food grade), growing medium in hydroponic gardens and plotted plants and landspreading [72, 73].

#### Trub

This is an unavoidable by-product obtained principally in the separator after the brewing process. It is formed of hops, inactive yeast, heavy fats and proteins. Currently this waste is mixed with spent grain and sent to animal feeding, which is the best alternative according to the FWMDT (Fig. 7).

On the other hand, while hops are typically considered inedible, some parts are actually edible. For example, hop shoots can be consumed by humans [74]. Ideally edible parts of the hops would be separated and used in food products and the remaining hops be sent to animal feeding. Yeast, fats and proteins could potentially be used in food products. As well as with spent grain, discussed previously, production of new food products was not selected by using the FWMDT because trub was considered inedible, as there is either no current consumer demand for the products described above or no technology available to undertake the processes required.

#### Applicability of the Categorization Process and the FWMDT

The FWMDT was proved to be useful to classify food waste generated at Molson Coors, as two types of waste were identified to be upgradeable: waste beer and filter waste could be managed in an alternative way in which more value would be obtained.

The assessment of some categories was complex for some food wastes, e.g. edibility for spent grain and waste beer. Spent grain was demonstrated to be edible, but as there is no market for this product for human consumption spent grain waste was consequently further classified as inedible. Research and investment to produce new food products from spent grain is encouraged, and when that takes place the categorization of spent grain will have to be amended. Waste beer was classified as eatable, however safety concerns regarding beer left in casks brought back from the food service sector must be overcome before the beer is reused. Should waste beer be considered safe for consumption but of low quality, ethical issues may arise regarding the benefits of using it for human consumption. Following the FWMDT, redistributing safe food for human consumption is always better from a sustainable point of view than any other alternative from the food waste hierarchy.

The feasibility to send food waste to animal feeding was also difficult to assess. It was found that when considering animal feeding for inedible, plant-based, single or mixed product not in contact with or containing animal-based products, non-catering waste (Fig. 7) each type of food waste should be analysed independently. For instance, trub can be sent for animal feeding but filter waste not because it contains diatomaceous earth which cannot be digested by animals.

Additionally, waste formed principally of yeast could not be strictly classified as plant-based or animal-based. The ‘microorganisms’ indicator was introduced for this reason, but in practice this was considered as plant-based material, since it is not under animal by-product regulations.

Molson Coors also generates a by-product from the mashing process, spent yeast, which is currently sold to a food company nearby to produce Marmite^®^, a food spread. Since this by-product is sold as planned by Molson Coors to produce a food product, it is not considered food waste according to the definition provided previously, and therefore is out of the scope of this work. If spent yeast were sent for any other use, it would be considered food waste and would have to be analysed using the FWMDT.

### Manufacturer of Meat Alternatives: Quorn Foods

This section categorizes the different types of food waste generated at Quorn Foods, a manufacturer of meat alternatives situated in Northern England. Two types of food waste were identified: food solid/slurry mix and food product returns, which account for 63 and 21 % of the total waste in the factory respectively. The rest of the waste is non-food materials such as cardboard, plastic, etc. The quantity of waste generated during a year is only conditional on the level of production: a relatively constant percentage of waste is generated per amount of final product manufactured. The different food waste types are listed and categorized in Table 2 and explained below.

**Table 2:** Types of food waste in Quorn Foods and their management alternatives

	Food solid/slurry mix	Food product returns
Edibility	Edible	Edible
State	Eatable	Eatable
Origin	Fungus*	Fungus*
Complexity	Mixed product	Mixed product
Animal-product presence	Not in contact with or containing meat, animal by-products or raw eggs	Not in contact with or containing meat, animal by-products or raw eggs
Treatment	Unprocessed	Processed
Packaging	Unpackaged	Separable from packaging
Packaging biodegradability	N/A	N/A
Stage of the supply chain	Non-catering waste	Non-catering waste
Current treatment	Animal feeding	Anaerobic digestion
Suggested alternative	Animal feeding	Redistribution for human consumption
Further possibilities	Production of foodstuff	N/A
Quantity	1000 t/year	≈360 t/year

The suggested alternative is based on the FWMDT presented in the Figs. 4–7. Possible alternative options from the food waste hierarchy are suggested as further possibilities when they are better than the suggested alternative. N/A means ‘not applicable’ or that the information is not necessary. * The ‘fungus’ indicator, from the origin stage, was considered as plant based

#### Food Solid/Slurry Mix

This category of waste includes products being lost through the production line: product falling from conveyor belts, trimmings, product stuck onto inner walls of the industrial equipment, etc. It has the same ingredients as the final product: fungus (mycoprotein), plant-based material, and animal-based products (egg albumen) in low proportions: 2–3 % by mass of the final product. It is an avoidable waste as it could be reduced or eliminated with more appropriate industrial equipment.

This waste was considered eatable, as it is generated only because of the inefficiency of the systems rather than to due to problems with the product. However, a more detailed analysis should be carried out to identify all different cases where this waste is generated and assess their state. If uneatable waste (e.g. spilled food onto the floor) is found, this should be classified as a different category of waste [75], although the new food waste management alternative for this waste according to the FWMDT would remain unchanged in this particular case: animal feeding.

Considering the previous comments, the most beneficial alternative according to the FWMDT (Fig. 5) is animal feeding, which is the option currently followed by the company. Unfortunately, this does not provide any economic income at present.

An investment in improvements in the industrial equipment would reduce the amount of food wasted in this category. Alternatively, the waste generated could be recovered and used to produce more final product.

#### Food Product Returns

Food product returns is the final product which cannot be sold to the final consumer for a number of reasons, including incorrect formulation, no traceability, packaging errors, etc. It has the same ingredients as the final product: fungus (mycoprotein), plant-based material, and animal-based products (egg albumen) in low proportions: 2–3 % by mass of the final product. It is an avoidable waste as it could be reduced or eliminated with more appropriate manufacturing practices.

This waste was considered eatable, as it corresponds to the final product. However, a more detailed analysis must be carried out before redistributing the food for human consumption in order to identify all different cases where this waste is generated and assess their state. If uneatable waste is found (e.g. its use-by date has passed), it must be classified as a different category of waste and this will allow a bespoke solution for this type of food waste. In this case, since the product is packaged, there is no risk of uneatable waste contaminating eatable waste.

Considering the previous comments, the most beneficial alternative is redistribution for human consumption, according to the FWMDT (Fig. 5). Currently the waste is separated from its packaging and sent to anaerobic digestion. The remaining packaging is used to produce refuse-derived fuel.

#### Applicability of the Categorization Process and the FWMDT

The FWMDT was proved to be useful to classify food waste generated at Quorn Foods, as one type of waste was identified to be upgradeable: food product returns could be managed in an alternative way in which more value would be obtained.

A more detailed analysis would be useful to identify sub-types of food waste and consequently the categorization process should be completed for all new food wastes found. This would provide a tailored waste management alternative for each type of food waste. For instance, if a final product for which the use-by date has passed is found, this could be named as ‘expired food product returns’ and its most appropriate waste management alternative would be anaerobic digestion, unlike the current generic ‘food product returns’ which should be redistributed.

Additionally, waste formed principally of fungus could not be strictly classified as plant-based or animal-based. The ‘fungus’ indicator was introduced for this reason, but in practice this was considered as plant-based material, since it is not covered by animal by-product regulations.

## Conclusions

The food waste categorization and management selection flowchart (i.e. the Food Waste Management Decision Tree) discussed in this paper facilitates the selection of the most sustainable food waste management alternative, with the objective of minimizing environmental impacts and maximising economic and social benefits. The categorization is intended to be easy to apply, facilitating identification of the type of food waste generated, and its link with the most appropriate food waste management alternative. This methodology has been illustrated with case studies from two large UK food and drink manufacturers. Their food waste types have been identified and their existing waste management practices compared to the proposed alternatives. It was found that a detailed breakdown of the types of food waste provides significantly better results than general itemisation, since bespoke solutions can be used for each food waste.

The analysis described can be applied to every type of food waste from every stage of the food supply chain. However, this methodology is expected to be more useful in the early stages (agricultural and manufacturing) of the food supply chain, where separate collection is generally carried out more effectively, than in the retailing and consumer stages where waste is often sent to municipal solid waste. Additionally, it is recommended to adapt the categorization to each food sector or business and include more waste management alternatives in the analysis (e.g. extraction of compounds of interest from food waste).

Unfortunately, the alternatives at the top of the food waste hierarchy are applicable to fewer food waste types than those at the bottom. Consequently, a range of solutions is required for a tailored treatment of each food waste type. A clear example of this is the reduction in the previously widespread use of food waste for animal feeding. This is due to stricter regulation that has resulted in fewer types of food waste that can be used to feed animals [76]. Health and safety concerns influence legislation on food waste management, but excessively zealous bans of food waste management options results in the unintended consequence that less advantageous alternatives are more commonly used. Regarding the animal feeding example, there are initiatives to change legislation and allow more types of food waste to be fed to animals [77].

The food waste categorization scheme is also useful for monitoring purposes. It provides an easy way to classify food waste in a business or a region to assess progress in management and sustainability and measure against other companies or areas. In order to do that, firstly a clear definition of food waste must be agreed, the boundaries of the system to analyse must be delimited, and afterwards the food waste types can be identified and quantified.

Evaluating the relative merits of waste management alternatives is a complex task. The factors determining which solution is more convenient are difficult to assess and sometimes even difficult to identify, including yields of the processes, proximity of waste management facilities, tax regulations, and demand for by-products, amongst many others. As a consequence, the waste hierarchy should be applied to every type of food waste identified independently, rather than to food waste as a whole, and undertake an exhaustive analysis for each food waste. To meet this challenge the authors are developing an analysis method and associated figures of merit to allow quantitative comparison of waste management alternatives, with a focus on environmental impacts, as an improvement over the current, qualitative approach.
